# Isotopic Niche Variation in a Higher Trophic Level Ectotherm: Highlighting the Role of Succulent Plants in Desert Food Webs

**DOI:** 10.1371/journal.pone.0126814

**Published:** 2015-05-14

**Authors:** Miguel Delibes, Ma Carmen Blazquez, Jose Maria Fedriani, Arsenio Granados, Laura Soriano, Antonio Delgado

**Affiliations:** 1 Department of Conservation Biology, Estación Biológica de Doñana, CSIC, Avda. Américo Vespucio s/n, 41092, Seville, Spain; 2 Centro Investigaciones Biológicas Noroeste, Avda. Politécnico Nacional 195, 23090, La Paz, B.C.S., Mexico; 3 Department of Ecological Modelling, Helmholtz Centre for Environmental Research GmbH—UFZ, Permoserstrasse 15, 04318, Leipzig, Germany; 4 Technical University of Lisbon, Institute of Agronomy, Centre for Applied Ecology, Tapada da Ajuda, 1349–017, Lisboa, Portugal; 5 Instituto Andaluz de Ciencias de la Tierra, CSIC-Universidad de Granada, Camino del Jueves s/n, 18100, Armilla, Granada, Spain; Universidad de Granada, SPAIN

## Abstract

Stable isotope analysis of animal tissues allows description of isotopic niches, whose axes in an n-dimensional space are the isotopic ratios, compared to a standard, of different isotope systems (e.g. δ^13^C, δ^15^N). Isotopic niches are informative about where an animal, population or species lives and about what it consumes. Here we describe inter- and intrapopulation isotopic niche (bidimensional δ^13^C-δ^15^N space) of the Orange-throated whiptail (*Aspidoscelis hyperythra*), an arthropodivorous small lizard, in ten localities of Baja California Sur (Mexico). These localities range from extreme arid to subtropical conditions. Between 13 and 20 individuals were sampled at each locality and 1 cm of tail-tip was collected for isotope analysis. As expected, interpopulation niche width variation was much larger than intrapopulation one. Besides, isotopic variation was not related to age, sex or individual size of lizards. This suggests geographic variation of the isotopic niche was related to changes in the basal resources that fuel the trophic web at each locality. The position of Bayesian isotope ellipses in the δ-space indicated that whiptails in more arid localities were enriched in ^13^C, suggesting most of the carbon they ingested came from CAM succulent plants (cacti, agaves) and in minor degree in C_4_ grasses. Contrarily, whiptails in subtropical areas were depleted in ^13^C, as they received more carbon from C_3_ scrubs and trees. Localities closer to sea-level tended to be enriched in ^15^N, but a clear influence of marine subsidies was detected only at individual level. The study contributes to identify the origin and pathways through which energy flows across the trophic webs of North American deserts.

## Introduction

The classical concept of ecological niche corresponds to a hypervolume in n-dimensional space, with the axes representing environmental variables (or resources) along which it is delimited the range where a species can survive (fundamental niche) or actually is present (realized niche) [1–2]. But historically practical difficulties to apply the niche concept to different species and circumstances [3] moved some ecologists to accept that, although the "niche is a central concept of ecology (…) we do not know exactly what it means" [4], reaching some of them (e.g. [5]) to recommend avoid the term niche in the scientific literature.

Recently, however, new methodological and technological approaches are renewing the concept of ecological niche and improving the ways to quantify it [6–7]. In particular, the use of stable isotope analysis (hereafter, SIA) on animal tissues is allowing describing isotopic niches, whose axes in a multidimensional space would be different isotopic signatures (e.g. δ^13^C or δ^15^N) [8]. Isotopic values can indicate what an animal had consumed (e.g. [9]) and some characteristics of the place or the habitat in which it lives or lived (e.g. [10]).

Isotopic signatures of primary producers are carried up to the highest levels in the food web with only minor changes. Thus, the geographic variation in the isotopic niche of generalist predators would reflect spatial changes in their food-webs [11]. For example, intraspecific variation in isotopic niche has been detected between predator populations of distant areas [12] and those relying on freshwater and marine food-webs [8]. At lower spatial scales, isotopic niche of particular populations will include individual variation in the ranges of prey consumed, but also intra-locality variation among food sources [13–14]. Consequently, interpopulation variation in isotopic niche across a species range can inform about the spatial patterns of isotopic variation in primary producers, while within-population variation can illustrate about individual patterns of resource use at local level.

We have compared the long-term isotopic niche of ten populations of Orange-throated whiptail (*Aspidoscelis hyperythra*; Sauria, Teiidae). *A*. *hyperythra* is an arthropodivorous small lizard that can be considered a good model of higher trophic-level consumer at the Baja California desert food-webs (see below). We used estimates of δ^13^C and δ^15^N in body tissues characterized by a low-turnover. Our main aim was to relate whiptail niche variation to geographic differences in the type of plants fuelling the communities and to individual (intrapopulation) variability in the use of resources.

The main condition to obtain informative results from SIA is to have enough variation in the isotopic composition of the primary resources [6]. In our study, we expected to find at least four sources of isotopic variation: a) at interpopulation scale, primary producers at different areas would be mainly succulent plants (many cacti and some agaves) or bushes and trees; both groups use different photosynthetic pathways and consequently their δ^13^C signatures are very different (see Methods and S1 Table); b) also, the food-webs of the areas closer to the sea could be more or less subsidized by nutrients from marine origin, which have contrasting δ^15^N signatures [15]; c) at intrapopulation scale, individuals can behave as specialist on different food sources at isotopic level (“individual niche”) [16]; for instance, home ranges of individual lizards vary in vegetation, soil type, humidity, etc.; this will make some individuals to rely more on arthropods feeding on succulents, while others will rely on those feeding on bushes; d) finally, at the localities with less complex communities it could be expected to find shorter food-chains, which could be detected at the isotopic composition of predators (mainly δ^15^N, that increases about 3 points upwards each trophic level [17–18]; however, this discrimination factor could be lesser in ectotherms, making difficult the assignation of trophic levels [19–20]). Based on these potential sources of variation we predict:

1. Interpopulation variation in the isotopic niche of our high-level predator will be greater than intrapopulation variation, mainly (but not only) due to the contrasting vegetation characteristics in our sampled localities.
2. The isotopic niche of each population will reflect the biotic and abiotic environmental conditions of the area. An increased importance of succulents as primary producers fuelling the food-web, detected as an enrichment of ^13^C (i.e. less-negative values of δ^13^C) and maybe higher δ^15^N, was expected at drier, more desert localities. Also, the probability of detect some influence of marine subsidies through an enrichment of ^15^N, should be higher at low altitudes and closer to the sea.
3. Individual (intrapopulation) variation in the isotopic niche should be high, as whiptails have reduced home ranges and thus each of them is dependent on the resources of a limited area in the “mosaic nature of the environment” [1]. Thus, they could behave as specialist although the species or population was generalist [14, 21]. However, we do not expect isotopic variation related to the sex or size of lizards, because they are very generalist arthropodivores, lack of apparent sexual dimorphism and use the same habitats all along their life (see below).

Until now, few studies have used small terrestrial ectotherms as model organisms for isotopic studies (but see [22–24]). Also, SIA have been relatively scarce in desert biomes [25]. To our knowledge, this is the first study evaluating geographical variation in the isotopic niche of a desert high-trophic level ectotherm. The results should be informative about the less-known relative importance of different plant types as producers fuelling animal communities in North American deserts.

## Study System, Material and Methods

### Model species

Orange-throated whiptails (herein whiptails) are small lizards (4–7 g in weight) native to Southwestern California (USA) and the peninsula and some islands of Baja California (Mexico). Their sexual dimorphism is scarce, being not possible to distinguish males from females at field conditions unless you catch them. They are non-territorial and forage actively during the day, searching in the leaf litter, the shallow soil and the low branches for a large variety of animal foods ([26–30], authors pers.obs.). Following [31], they should be considered generalist arthropodivores. Their individual hunting range is small, about 445 m^2^ [32], while the mean distance between recaptures of the same individual approached 11 m [28]. Because of their foraging behaviour (they are active searchers and not ambushers [30]) and their wide food-spectrum, they can be considered suitable “samplers” of the local community of arthropods, which includes different trophic roles (herbivores, detritivores, predators, parasitoids). Therefore, the species is a good model of higher-level consumer in the desert community.

### Study area

The study has been carried out in the southernmost quarter of the Baja California peninsula, approximately between 23°N-26°N in latitude and 109°W-112°W in longitude. Baja California is a volcanic fringe of land running from north-west to south-east, 1300 km long and, on the average, about 100 km wide. Most of the study area is considered “thermotropical” [33], with annual mean temperature of about 23°C. Rains, concentrated mainly in the summer, are very scarce, increasing towards the south and in altitude (we did not sampled whiptails above 500 m; see Table 1 for characteristics of each locality).

**Table 1:** Main characteristics of the ten sampled localities (ordered from South to North).

Locality	Coord. (N, W)	Coastline dist. (km)	Altitude (m.a.s.l)	Rainfall (mm)	Veget.type	δ ^13^C‰(n)	δ ^15^N‰(n)
**CSL**	22.92360109.9774	4.22	173	239	1	-17.87 ± 0.36 (16)	10.32 ± 0.26 (16)
**MIG**	23.06735110.0984	0.98	45	202	2	-21.29 ± 0.32 (19)	11.16 ± 0.33 (19)
**CNAR**	23.24340109.7461	21.74	209	413	1	-25.22 ± 0.70 (19)	10.73 ± 0.13 (20)
**RIB**	23.56870109.5598	2.72	27	217	1	-20.33 ± 0.14 (20)	12.81 ± 0.48 (20)
**BART**	23.74020109.8694	15.97	447	397	1	-21.89 ± 0.13 (19)	7.83 ± 0.27 (19)
**INO**	23.77415110.6611	1.98	13	93	4	-17.89 ± 0.27 (19)	14.31 ± 0.21 (19)
**KM 83**	24.26863110.9555	19.69	153	129	2	-18.26 ± 0.33 (20)	12.58 ± 0.21 (20)
**TECO**	24.34629110.2862	0.53	31	217	3	-18.47 ± 0.39 (15)	7.74 ± 0.30 (17)
**IHU**	24.97434111.4075	51.63	134	160	2	-17.03 ± 0.43 (13)	12.09 ± 0.40 (13)
**BAJO**	26.08441111.3253	0.09	8	130	2	-21.53 ± 0.28 (20)	13.13 ± 0.34 (20)

Sample sizes (n) and levels of δ ^13^C and δ ^15^N (mean + SE) correspond to lizard tail tips for each locality. Shortest distance to the coastline and average altitude were obtained from a GIS of the area, average annual rainfall was estimated from WORLDCLIM Ver. 1.2 (http://www.worldclim.org) and vegetation type was assigned from 1:250000 charts of [77]. Vegetation types: 1, deciduous dry forest; 2, desert scrubs (mostly Cactaceae); 3, halophyte scrubs; 4, fog desert shrub (mostly Cactaceae, but with humidity from the sea and lichens).

Very arid desert conditions predominate in the north of the area, while subtropical conditions influence the south [34]. Many species of cacti are present in the desert, especially giant cardons (*Pachycereus pringlei*), which frequently characterize the landscape, and *Opuntia* spp., *Stenocereus* spp., *Ferocactus* spp., *Mammillaria* spp., etc.; different agaves (*Agave* spp.) and Joshua trees (*Yucca* spp.) are common too; trees are scarce (some legume trees such as *Prosopis* sp. and *Lysiloma candida* at the usually dry riverbeds) but there are shrubs, such as *Larrea divaricata* and *Fouquieria* spp. On the other hand, at the subtropical thorny forest of the south, succulents are also abundant, mainly at low altitude, but woody shrubs, large bushes and deciduous trees, including *Bursera* spp., *Lysiloma divaricata*, *Tecoma stans*, *Cyrtocarpa edulis*, *Jatropha cinerea*, *Cercidium* spp., *Caesalpinia* spp., *Mimosa xantii*, etc., predominate [35]. Near the shore there are some mangrove species (*Rhizophora mangle*, *Laguncularia racemosa*, *Avicennia germinans*).

Succulents, trees, shrubs and grasses use several photosynthetic pathways, which have an important effect on their isotopic signatures [36]: C_3_ plants fix CO_2_ using the enzyme ribulose bisphosphate carboxylase and typically have δ^13^C values rounding -28‰ (-26.43 + 1.75, mean + SD, in our samples; S1 Table); C_4_ plants take up initially CO_2_ by carboxylation of phosphoenolpyruvate, having δ^13^C signatures around -14‰ (-14.86 in our sample; S1 Table); CAM plants refer to Crassulacean Acid Metabolism and have δ^13^C signatures rather similar to C_4_ plants (-13.91 + 1.24 in our sample; S1 Table). While cacti and agaves (and at least one succulent Euphorbiaceae species) are photosynthetic CAM plants in Baja California, trees are obligate C_3_ and, according our data (see S1 Table), all woody shrubs would be C_3_ plants. Most grasses are potential C_4_ plants (S1 Table), but they are spatially and temporally very scarce in the area, except locally following summer rains, which do not reach all areas every year.

### Whiptail capture and sampling

**Fig 1:**
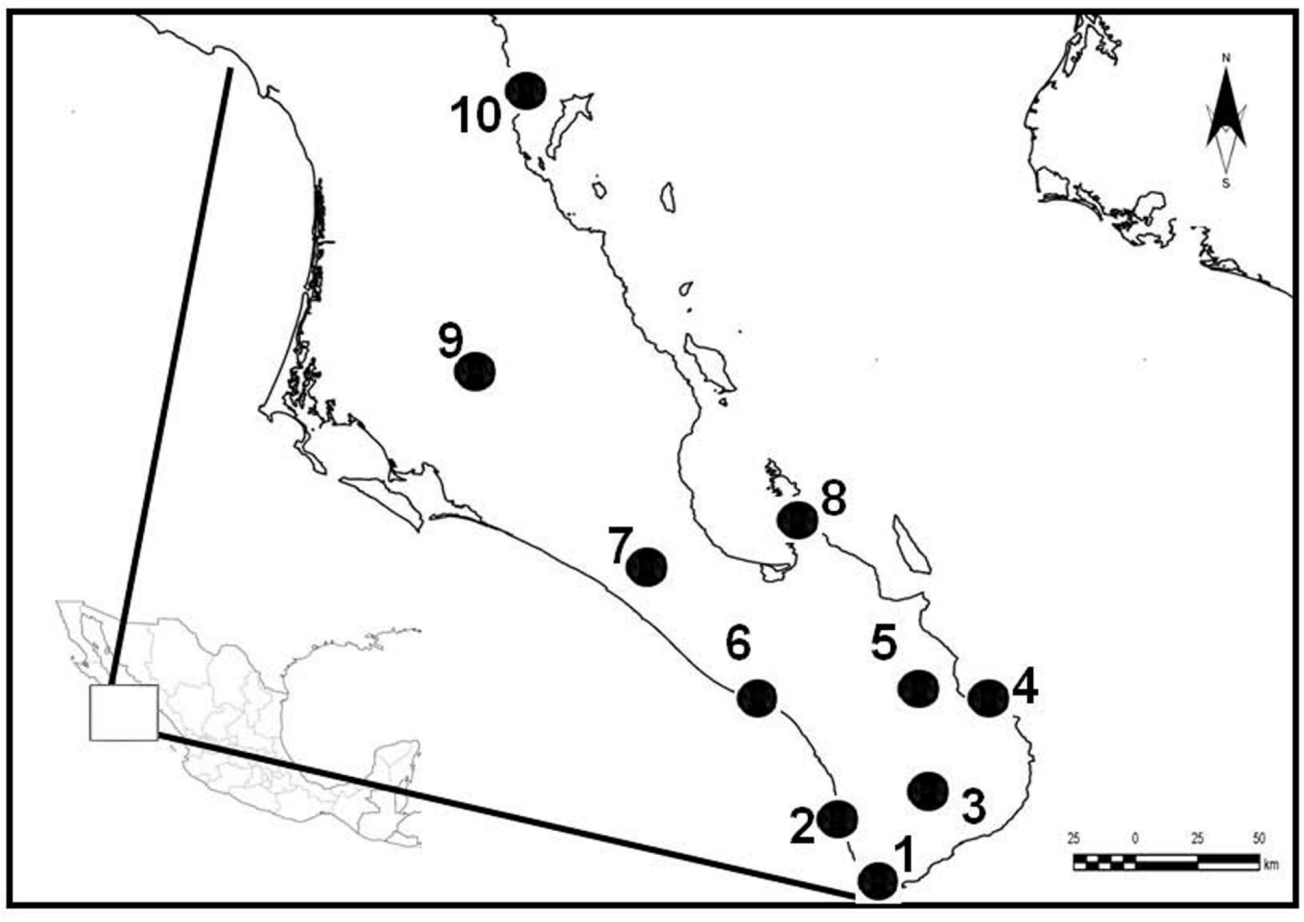
Map of the study area showing the sampled localities. 1: Cabo San Lucas (CSL). 2: Migriño (MIG). 3: Camino de los Naranjos (CNAR). 4: La Ribera (RIB). 5; San Bartolo (BART). 6: Los Inocentes (INO). 7: Kilómetro 83 (KM83). 8: El Tecolote (TECO). 9: Presa de Ihuazil (IHU). 10: El Bajo (BAJO).

In different periods from September 2005 to February 2010, between 13 and 20 whiptails were captured and sampled in each of ten localities well-spaced in the area (Fig 1). These localities vary widely in vegetation type, annual rainfall,altitude and distance to the sea (see Table 1). To avoid killing whiptails, we snared them with nooses across their head, by a fishing rod with a thread loop at the tip. Immediately after capture, each individual was sampled by cutting about 1 cm of the tail tip using a scalpel disinfected with ethanol, and released within a few minutes, of all them run immediately. Individuals with obviously regenerating tail were overlooked. Tail-clipping is frequently used in the study of reptiles, as it is considered a nondestructive sampling technique (e.g. [37]). Besides, at field conditions it is an efficient method for preventing re-sampling. Whiptails have the ability for tail autotomy and posterior regeneration, so the loss of just the tail tip should not be too stressful for them (see [38]). Tail tip clips were used for isotopic analyses.

All animals were captured on open public land and close to roads or villages, where free access and transit was allowed. Because the localities CNAR, MIG, BART and RIB are in the buffer zone of the Biosphere Reserve “Sierra de la Laguna” and *Aspidoscelis hyperythra* is an endemic species catalogued in Mexico as threatened, to obtain the permit from the authorities we send them previously a detailed protocol. Permission for the complete protocol (capture, sampling of tail, and releasing) was obtained from the environmental authority in Mexico (D.G. Vida Silvestre, Secretaría de Medio Ambiente y Recursos Naturales, México, permit # 11311; see Acknowledgments section), who is in charge of the ethical considerations. There is not a formal Institutional Animal Care and Use Committee.

### Stable isotopic analyses

Isotope measurements were carried out at the Stable Isotope Laboratory of the Instituto Andaluz de Ciencias de la Tierra (CSIC-UGR, Granada, Spain). Organic matter (lizard tail tips and plant tissues) was homogenized and later analyzed for the isotopic composition of nitrogen and carbon by means of a Carlo Elba NC1500 (Milan, Italy) elemental analyzer on line with a Delta Plus XL (ThermoQuest, Bremen, Germany) mass spectrometer (EA-IRMS). Because practically lizard tail tip does not content lipids [39] and little is known in detail about isotopic routing in Lacertidae, we did not extract lipid from our samples (but see [40]). The stable composition of samples is reported as δ values per mil:

δ=(Rsample/Rstandard−1)*1000

Where *R* = 13*C*/12*C for δ*13*C values and R* = 15*N*/14*N* for δ^15^N values.

Commercial CO_2_ and N_2_ were used as the internal standard for the carbon and nitrogen isotopic analyses. For carbon, two internal standards of -30.63 ‰ and -11.65 ‰ (Vienna Pee Dee Belemnite; VPDB) were analyzed every 10 samples. For nitrogen, two internal standards of -1.02 ‰ and +16.01 ‰ (AIR) were used. Precision calculated, after correction of the mass spectrometer daily drift, from standards systematically interspersed in analytical batches was better than ± 0.1‰ for both δ^13^C andδ^15^N.

All the individual isotopic values can be consulted in additional supporting data (S2 Table)

### Isotopic niche metrics

Isotopic niche characteristics were described with quantitative metrics developed by [41], which used mean δ^13^C and mean δ^15^N of all species in a given community. In order to adapt their community level metrics to a population level, able to encompass intra and interpopulation variation, we did not use means, but the values of δ^13^C and δ^15^N of all individuals sampled in each population. For each population we calculated:

1. Total Area (TA) of the convex hull encompassing all points, which can be considered a measure of population niche width. Nevertheless, because this estimator is very dependent of sample size (e.g. [42]), we also calculated:
2. Bayesian Standard Ellipse Area (SEA_B_), bootstrapping data (n = 10000). Standard ellipse (SEA) contains approximately 40% of the points and it is a measure of the mean core population niche, being to bivariate data as standard deviation is to univariate data [43]. It provides a good estimate of the population niche area, but with tendency to underestimate at small size samples. SEA_B_ nullify this bias, allowing comparisons among populations widely differing in size sample. Mean areas and the low and upper 95% credible limits will be shown. Additionally, we calculated:
3. Carbon range (CR) and Nitrogen range (NR), corresponding to the distance between the two individuals with the highest and the lowest δ^13^C and δ^15^N values within each population; they estimate the total carbon and nitrogen range exploited by each population.
4. Mean Distance to Centroid (CD), calculated as the mean Euclidean distance of each individual of a population to the δ^13^C-δ^15^N centroid for that population; it is an estimator of the population isotopic diversity.
5. Mean Nearest Neighbor Distance (MNND) that reflects the packing of the individuals in the two-dimensional isotopic space.

All calculations were made in R using the SIAR package [44].

### Geographic variation analysis

Besides the graphical representation of the ellipses characterizing isotopic niches, differences among localities in δ^13^C and δ^15^N levels were analyzed fitting general linear mixed models using Proc Glimmix in SAS [45], including the sampling date as a random factor. To evaluate our prediction that δ^13^C local levels would relate to aridity and those of δ^15^N to coastline distance, altitude, and aridity, we regressed their mean values across localities. In addition to evaluating our predictions, we also evaluated the effect of other five ecological correlates or potentially confounding factors: season (i.e. proportion of individuals sampled in summer-autumn), age (proportion of sampled adults), sex (proportion of adult females when juveniles were not considered), size (snow-went length) and occurrence of regenerated tail (proportion of sampled individuals with apparently regenerated tail). Because of departures from normality and the presence of outliers, we used robust regression [46] rather than least-squares regression. Robust fits are minimally influenced by outliers in the independent variable space, in the response space, or in both. Analyses were performed by using robust MM-regression procedure available in S-Plus 6 [47], and the significance of each variable was assessed by using robust F-tests (F_R_).

Given that spatial autocorrelation could inflate Type I statistical error, we evaluated whether residuals from significant robust fits for mean population values were spatially autocorrelated. To this end, we used Moran’s I mark-correlation function [48] which allowed us to investigate how the residuals of two populations separated by distance r differ from their expected value under a null model of not spatial autocorrelation. It ranges from −1 (indicating perfect dispersion) to +1 (perfect correlation). Spatial autocorrelation analyses were conducted with the software *Programita* [48] available at www.Programita.com.

## Results

### Isotopic niche descriptors

**Fig 2:**
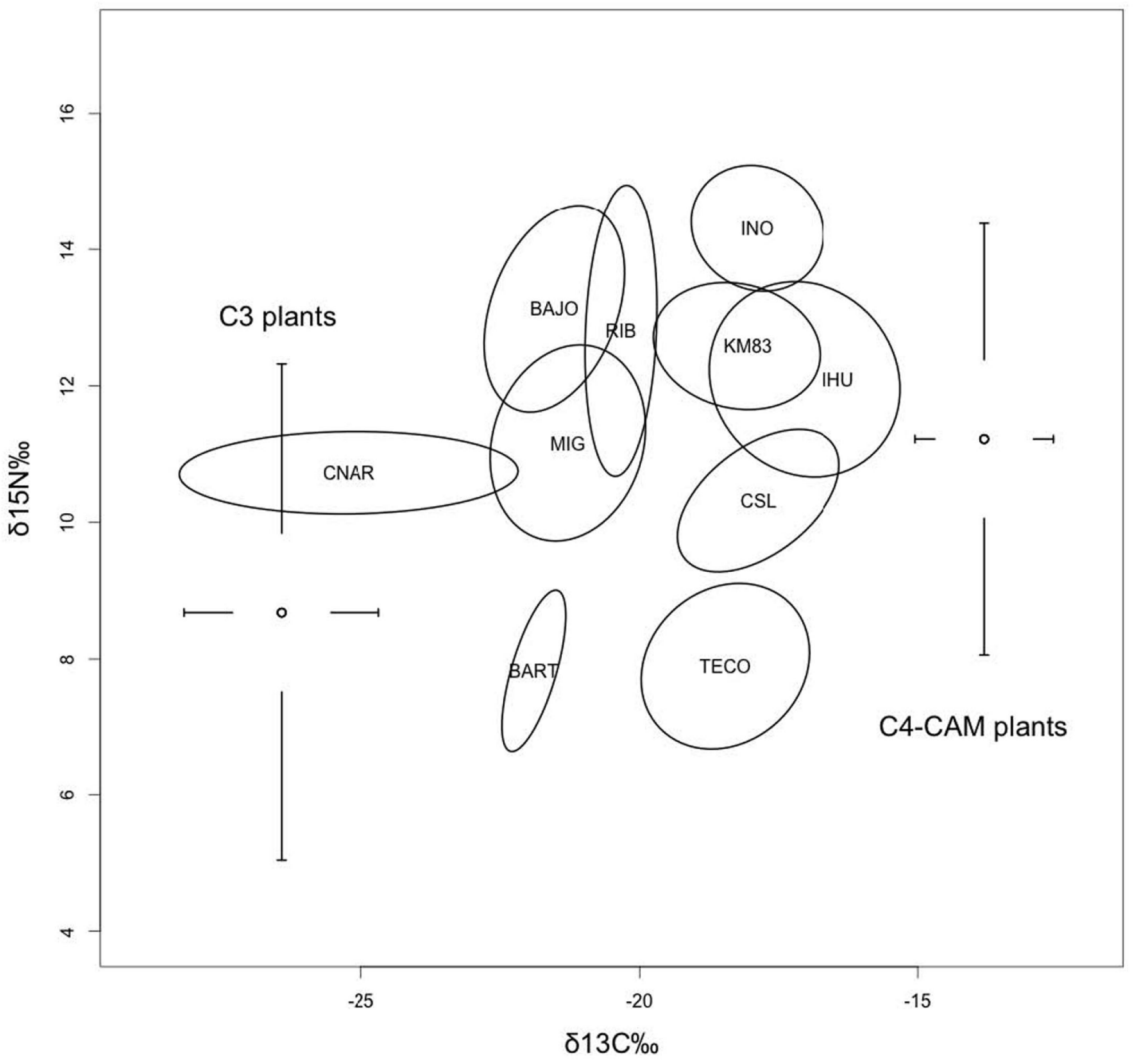
Stable isotope standard ellipses of whiptails for each of ten studied localities in southern Baja California (Mexico). Ranges and mean values of δ^15^N and δ^13^C of C_3_ y C_4_-CAM plants sampled in the whole area are also shown. Localities are named according to Table 1.

Our localities differ widely in biotic and abiotic conditions, as well as in the average isotopic values of their whiptail tissues (Table 1). Also, there is a substantial geographic variation in the isotopic metrics (Table 2) and the position of population ellipses in the bi-dimensional δ^13^C-δ^15^N space (Fig 2).

**Table 2:** Stable isotope niche metrics for each lizard population and the whole sample.

Populations	TA	SEA_B_ (95% limits)	CR	NR	CD	MNND	n
**CSL**	11.15	4.40 (4.38–4.42)	5.36	3.53	1.51	0.64	16
**MIG**	17.42	6.26 (6.23–6.29)	5.93	5.47	1.73	0.63	19
**CNAR**	12.96	6.35 (6.32–6.38)	8.52	2.01	2.75	0.62	19
**RIB**	13.08	4.71 (4.69–4.73)	2.71	7.26	1.70	0.44	20
**BART**	4.69	2.09 (2.08–2.10)	2.78	4.45	1.03	0.30	19
**INO**	10.13	3.63 (3.61–3.64)	6.51	2.77	1.14	0.54	19
**KM 83**	12.74	4.50 (4.48–4.52)	6.51	3.68	1.41	0.66	20
**TECO**	13.22	5.78 (5.75–5.81)	4.68	3.64	1.72	0.76	15
**IHU**	19.28	7.58 (7.54–7.62)	6.29	4.66	1.92	1.01	13
**BAJO**	18.66	5.76 (5.75–5.79)	5.19	6.88	1.57	0.70	20
TOTAL	112.0	21.03 (20.99–21.06)	14.2	12.1	3.28	0.39	180

TA = Total area of convex hull; SEA_B_ = Bayesian standard ellipse area; CR = δ^13^C range; NR = δ^15^N range; CR = Mean distance to centroid; MNND = Mean nearest neighbour distance; n = bivariate sample size.

As expected, niche area was much broader at the species than at the population level, both for Total Area (TA) and for Bayesian Standard Ellipse Area (SEA_B_) (Table 2). Specifically, SEA_B_ at the species level was, on average, five times larger than at the population level, and up to ten times larger as compared with the smallest population value (BART). That suggests the wide whiptail species isotopic niche results in fact from the sum of thinner, more specialized, local niches. At population level, large niches as estimated by TA (e.g. IHU, MIG) seemed to the more related to simultaneously high values in Carbon Range (CR) and Nitrogen Range (NR) than to extreme values in any of them (e.g. CNAR, RIB).

Considering all individuals, CR and NR were rather large, indicating that whiptails use a large range of isotopic resources. δ^13^C ranged from -28.67‰ to -14.44‰ (coefficient of variation [CV] = 14.1%), encompassing the whole range of the plants sampled in the area (in fact, some vegetal source in CNAR, at least, must be more depleted in δ^13^C than any of the plant species represented in S1 Table). δ^15^N ranged between 5.7‰ and 17.8‰ (CV = 21.6%), inside the range of plants (C_3_ plants, mean + SD = 8.68 + 3.64; C_4_-CAM plants, 11.22 + 3.16). CR and NR varied widely among populations. The highest CR corresponded to CNAR, just the population more depleted in ^13^C on the average. Also, the highest NR corresponded to RIB, the second population more enriched on the average in δ^15^N.

Although no precisely, at population level, the metrics related to isotopic diversity (Mean Distance to Centroid, CD) and packaging (Mean Nearest Neighbor Distance, MNND) were related to estimators of niche width. For instance, the highest CD corresponded to CNAR, the population having a largest CR, and the lowest one to BART, the population having smallest TA, SEA_B_ and MNND. Also, the highest MNND corresponded to IHU, the population having largest TA and SEA_B_.

With respect to the position of the ellipses in relation to δ^13^C axis (Fig 2), CNAR ellipse had the lowest central value (-25.22), while the more enriched was IHU ellipse (-17.03). En relation to δ^15^N, the lowest central value corresponded to TECO population (7.74) and the highest to INO (14.31). δ^13^C and δ^15^N tended to be positively related (see below).

### Geographical variation

**Fig 3:**
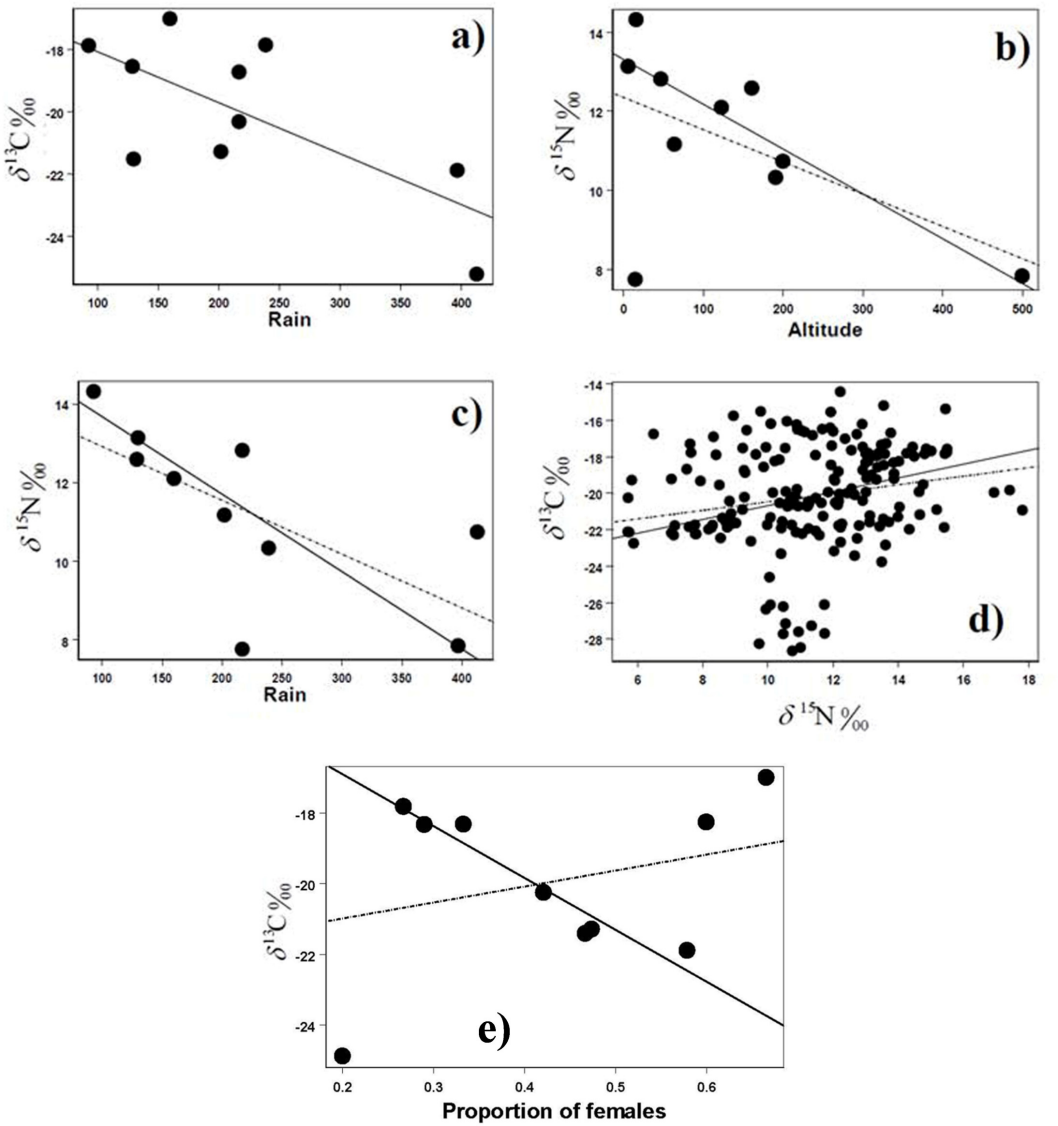
Relationships between isotopic values and several variables across the ten sampled localities in southern Baja California (Mexico). (A) Negative relationship between average values of δ^13^C (a) or δ^15^N (C) in whiptail tissues and the estimated annual rainfall (B) Negative relationship between average δ^15^N values and local altitude. (D) Positive relationship of δ^13^C and δ^15^N values across individual lizards (n = 180). Note that robust regression (continuous line) often differs somewhat from least-squares regression (dotted line). The arrows in charts b and c point to TECO sampling site. (E) Negative relationship between average values of δ^13^C in whiptail tissues and the proportion of females in the ten sampled localities. Note that robust regression (continuous line) has even different sign (i.e. negative) than that estimated by standard least-squares regression (i.e. positive; dotted line).

We did not find any relationship of δ^15^N with non-geographical factors such as sampling season, age, sex, size, or the occurrence of a regenerated tail (*P* > 0.166; see Table 3). In the case of δ^13^C, sampling season, age, size, or the occurrence of a regenerated tail did not have any significant effect (*P* > 0.074; Table 3). However, δ^13^C was negatively related with the proportion of females in population samples (*P* < 0.001; Fig 3E). This effect, again, was not revealed by standard least-square regression (F_1, 8_ = 0.096, *P* = 0.419).

**Table 3:** Main results from robust regressions for the relationships of δ^15^N and δ^13^C in *Aspidoscelis hyperythra* tissue and different ecological correlates.

	δ^15^N			δ^13^C		
	d.f.	F_R_	*P*	d.f.	F_R_	*P*
**Season**	1	1.405	0.225	1	1.871	0.161
**Age**	1	0.166	0.676	1	1.250	0.252
**Sex**	1	0.002	0.967	1	13.19	**0.001**
**SVL**	1	0.065	0.794	1	0.327	0.558
**Regenerated tail**	1	1.830	0.166	1	3.033	0.074

Season: proportion of individuals sampled in summer; Age: proportion of sampled adults; Sex: proportion of adult females when juveniles were not considered; SVL, Snout-vent length; Regenerated tail: proportion of sampled individuals with clear sign of regenerated tail. Significant result (*P* < 0.05) in boldface.

Contrarily, whiptails in the studied localities strongly differed in δ^13^C value (F_9, 163_ = 34.65, *P* < 0.0001). Average local values of δ^13^C tended to be higher in more desert areas, i.e. there was a significant negative correlation between average δ^13^C in each locality and annual average rainfall (F_R_ = 4.72, d.f. = 1, *P* < 0.05; Fig 3A). We also found strong differences in δ^15^N among localities (F_9, 165_ = 62.17, *P* < 0.0001). There was a significant negative relationship between local elevations and δ^15^N average values (F_R_ = 11.97, d.f. = 1, *P* < 0.001; Fig 3B), suggesting that lizards living closer to sea level (RIB, BAJO, INO) tended to include a higher proportion of heavier N in their tissues. Interestingly, this relationship turned no-significant (F_1,8_ = 3.57, *P* = 0.096) when using standard least-squared regression, likely because in contrast with robust regression it does not account for the effect of outliers such as the locality TECO (Fig 3B). Indeed, this locality shows the lowest δ^15^N value, although it is located close to the sea (also, it was the only one with halophyte vegetation; Table 1).

As expected, average δ^15^N values were negatively related to average annual rainfall (F_R_ = 11.43, d.f. = 1, *P* < 0.001; Fig 3C), suggesting that increased local aridity enhanced δ ^15^N levels (INO, IHU, Km 83). Contrary to expectations, no relationship was found between local levels of δ ^15^N and distance to coastline (*P* = 0.959).

There was not any significant relationship between average values of δ ^13^C andδ^15^N across localities (F_R_ = 0.32, d.f. = 1, *P* = 0.564). However, when considering individual whiptails as sampling unit (n = 180), there was a significant positive linear relationship between δ^13^C and δ^15^N (F_R_ = 5.47, d.f. = 1, *P* < 0.05; Fig 3D).

To evaluate a potential spatial autocorrelation in our data, we calculated Moran’s I at a range of spatial scales for the residuals of the three significant relationships concerning mean population values (i.e. δ^15^N *vs*. rainfall, δ^15^N *vs*. altitude, and δ^13^C *vs*. rainfall; Fig 3). In the three cases, observed Moran's I values were always within the simulation envelops (S1 Fig), indicating a lack of spatial autocorrelation in the residuals.

## Discussion

### Use of tail tip clips as samples

Tail tip clips from ectotherm vertebrates are frequently used as non-lethal sources of material for genetic (e.g. [49]), toxicological (e.g. [50]) and isotopic (e.g. [37,51]) studies. However, it must be considered that, besides diet, a large number of physiological, ecological and methodological factors generate variation in the isotopic signature of animal tissues [52, 25]. In particular, replacement rates of nutrient pools in each particular animal tissue do vary [53–54]. We have sampled the distal 1 cm of tail tip, which is made mainly by scales, skin, and bone, presumably tissues with low turnover; in addition, carbon incorporation rates in ectotherms are slower than those in endothermic vertebrates [55]. All this should minimize the effect of potential short-term variations in diet in our study (e.g, [24]). Lack of differences between pre- and post-summer whiptail samples in both δ^13^C (*P* = 0.378) and δ^15^N (*P* = 0.083) values supports this idea. However, some uncertainty regarding exact whiptail tail tip turnover time does exits, although it should not change our conclusions. Besides, in aquatic snakes it has been proved that scale clips and tail clips of the same individual do not differ substantially in isotopic composition ([56]). We assume tail tip stable isotope composition integrates the diet of whiptails along a time-span of several months.

### The wide isotopic niche of Orange-throated whiptail

For all-data pooled, the values of metrics related to isotope niche width, such as TA, SEA_B_, CR y NR, indicate a large niche area for the species. Practically the whole range of isotope variation in plants of the area was encompassed by CR and NR at species level, confirming Orange-throated whiptails are able to cope with a large variety of resources. However, as we expected, this large niche at the level of species seems to be due to the sum of smaller niches at population and subpopulation levels [7, 14].

Hutchinson [2] suggested the niche of a species has sceno-poetic and bionomic dimensions. The sceno-poetic axes refer mainly to the bioclimatic stage in which a species performs (i.e. where it lives), whereas the bionomic axes refer to the resources it uses (i.e. how it behaves). The distinction is particularly relevant in the analysis of isotopic niches, as isotopic signatures of resources otherwise similar change geographically [6]. Our results strongly suggest that the broad isotopic niche of *Aspidoscelis hyperythra* is mainly due to its sceno-poetic dimensions, i.e. to changes related to the large distribution range of the species. To begin, we have discarded a possible influence of most of non-geographical factors, such as season and whiptail size, age and length (although our results indicate that the specific sex composition of samples could bias results and thus caution is needed in interpreting trends from samples with different gender proportions).

Geographical variation in predator or competitor pressure could generate geographic changes into the realized isotopic niche of a species, as proved by [57]. However, the spectrum of predators and competitors of *Aspidoscelis hyperythra* must be rather similar in all our localities. Hence, we suspect the variation we found was most related to geographical changes in plant communities. Reinforcing this idea is the fact that the position of the population ellipses in the isotopic δspace can be related in a predictable way to geographical and environmental factors affecting plants (see below).

Whiptails are high-level generalist consumers, depredating on phytophagous invertebrates such as termites and on predators such as spiders. Unfortunately, we have not isotope data for arthropods at each locality. Thus, we cannot transform the isotopic niche in a trophic niche (see [6]) by applying the mixing models frequently used in the literature (see revisions in [25, 54]). However, by analyzing the position of population ellipses in the δspace we can infer the relative importance in the trophic web of producers with different isotopic signatures. For instance, according our predictions more arid and drier localities showed enriched average δ^13^C values, while subtropical localities showed depleted values. These results strongly suggests that in more desert localities (e.g., INO, KM83, IHU, TECO; Table 1, Fig 2) succulent plants and/or C_4_ grasses contribute most of the carbon circulating in the trophic web, while in more humid localities (e.g., BAR, RIB, MIG; Table 1) carbon would come both from succulents and C_3_ plants; only in CNAR, the locality receiving more rainfall, most of the carbon in the trophic web seemed to come from C_3_ scrubs and trees (Table 1, Fig 2).

### Variation in δ^13^C: the role of succulents in Baja California desert food webs

Isotopic signatures of C_4_ and CAM plants are very similar, although on the average CAM plants were enriched in ^15^N (S1 Table). Would be C_4_ grasses or CAM succulents the plants supporting high-level consumers in our desert food webs? Perhaps because most previous studies in dry areas took place in grasslands, many authors usually ascribed the nutrients labelled by less negative δ^13^C values to C_4_ grasses, neglecting the potential role of succulents (e.g. [58–60, 24]). However, as stated by [61], cacti and agaves can reach high densities and relative biomass in North America arid areas (e.g. until ~56% of seasonal above-ground biomass [62]) and several studies unequivocally showed that heavier carbon used by some desert consumers derived almost exclusively from cacti [61, 63–64]. In our study area, several evidences suggest that most of the nutrients enriched in δ^13^C had their origin in cacti rather than in C_4_ grasses. Succulents are dominant at the landscape (the study area includes the region of North America with more Cactaceae [65]), ground among bushes is usually barren, and frequently we saw no grasses in many of our ten sampling localities (S2 Fig). Besides, cacti feed a lot of arthropods [66] and we captured many lizards while foraging around and under dead cacti. In fact, a study in different localities at the centre of our study area showed that whiptails foraged mainly under the tree *Jatropha cinerea* and the cacti *Stenocereus gummosus* and *Cylindropuntia cholla* [67]. Also, the high δ^15^N values detected in the tissues of most whiptails seemed to be closer to those of succulents than to those of C_4_ gramineae in the area (S1 Table). Thus, our study suggests, as previously suspected, that the production by succulents is essential for animal communities in the Baja California desert.

### Variation in δ^15^N: Are there marine subsidies?

The interpretation of isotopic nitrogen levels in lizard tissues is more complicated, as many factors influence them [68–69, 18]. Variability of δ^15^N in plants may be high, mainly due to soil nitrogen cycling and plant physiology (see S1 Table). Besides, δ^15^N increases with the trophic level, and thus animals higher at the food chain tend to have higher δ^15^N values. Also, body condition affects the levels of isotopic nitrogen, as δ^15^N increases in starving animals [70] (but see [71]). Finally, at least in endotherms water stress does increase the proportion of δ ^15^N in animal tissues [72, 18].

However, most of these factors influencing δ^15^N can be considered minor ones when compared with the influence of marine subsidies at the food web. For instance, working with *Uta stansburiana* (another arthropodivorous lizard) at the north of the Gulf of California, it was found that δ^15^N reached about 28.8 ‰ in individuals captured on islands subsidized by seabird guano, 17.9 ‰ in those of coastal areas without birds, and only 13.0 ‰ in lizards of inland areas [15]. Considering these values and those of the plants at our study area (S1 Table), a clear influence of marine subsidies cannot be recognized in none or our localities. This was expected, because the area of marine influence is rather thin (about 50 m; [15]) and we extended our capture grounds several hundreds meters inland, with the exception of BAJO). However, we have found a significant relation between the average levels of δ^15^N and the altitude above sea level (Fig 2), suggesting some marine influence would be possible at lower localities (via insects feeding on marine products landed by people, mainly shells and fish remains, or on shore wrack, or receiving dust and aerosols [73]).

Our results in relation to δ^15^N could be also partially explained by the isotopic composition of plants and the effects of water stress on the nitrogen-isotope ratios in animal tissues. Thus, the negative relationship between δ^15^N and average rain (Fig 3C) could be expected. Also, the positive weak linear relationship between δ^13^C and δ^15^N values across individuals (Fig 3D) had been previously found for different species of endotherms, including humans [74, 18], and across species guilds [75].

### Intrapopulation isotopic variation

We have discarded an influence of age, size and other individual conditions. However, we have detected a rather high intra-local variation (individual niche [16, 76]) at least in some populations. This was reflected in the values of Total Area of the convex hull (TA), Carbon and Nitrogen ranges (CR and NR), Mean Distance to Centroid (CD) and other metrics (Table 2). We were not able to detect any general trend among localities, but it seems that the populations where more diversity of resources was expected (i.e. those having simultaneously high abundances of C_3_ and CAM plants), were those showing larger variation. For example, CNAR is the population more influenced by subtropical C_3_ plants and also it is the only locality covering more than 50% of the total CR (Table 2); high CR in CNAR can be explained because most (13 of 19; values not shown) whiptails were very depleted in δ^13^C, suggesting they relied on C_3_ plants derived resources, while 6 individuals enriched in δ^13^C apparently relied more on C_4_-CAM plants derived resources. In the same way, the two localities with higher variation in δ^15^N (RIB and BAJO, each of them covering more than 50% of the total NR; Table 2) were those placed lowest on sea level, where some influence of marine subsidies would be possible; in RIB, 17 individual whiptails had δ^15^N values under 13.0 ‰, but three others surpassed 17.0 ‰ (values not shown), suggesting this individuals had an atypical diet more influenced by the sea. This intralocality variation was predictable because microhabitat features (e.g. presence of a dead cardon or a C_3_ tree, availability of fishermen waste, etc.) can influence severely the isotopic signatures of available arthropods and thus whiptails.

### Concluding notes

This is the first study concerning the geographic variation in the isotopic composition of body tissues of a desert higher-level-trophic ectotherm. Trophic webs in North American desert ecosystems are rather complex [31], but using stable isotopes as technical tool and an arthropodivorous lizard as model, we showed that in Baja California Sur cacti and agaves (CAM plants), and likely to a lower extent C_4_ grasses, have a large importance in the supply of nutrients to the higher levels of the food webs, particularly in the more arid localities. Instead, the contribution of C_3_ shrubs and trees was important in southern localities with more rain and subtropical vegetation. A clear influence of marine subsidies has not been detected in any locality. Our study contributes to identify the origin and pathways through which energy flows in desert ecosystems, revealing overlooked food resources (i.e. cacti) with potential chief effects on the structure and dynamic of animal populations.

## Supporting Information

S1 Fig Spatial autocorrelation (measured as Moran´s I) for the residual of the three significant robust fits. A), δ15N and rainfall; (B), δ15N and altitude; (C), δ13C and rainfall.
(TIF)

S2 Fig The study area and lizards. **(**A) Landscape at a subtropical shrubland in the Southern of the study area; (B) Landscape at the dry desert in the North of the study area, with abundance of succulents; (C) a Whiptail capturing an Orthoptera in the South; (D) a whiptail on the Northern desert. All the pictures by M. Delibes.
(TIF)

S1 Table Values of δ15N and δ^13^C in sampled C3, CAM and C4 plants collected at different localities of the study area in southern Baja California (Mexico). (DOC)

S2 Table Values of δ15N and δ^13^C in each individual lizard sampled. The identification label corresponds to each population.
(XLS)

## References

[1] Hutchinson GE (1957) Concluding remarks. Cold Spring Harbor Symp Quantitative Biology 22: 415–427.

[2] Hutchinson GE (1978) An introduction to population ecology Hew Haven, Connecticut: Yale University Press. 271p.

[3] Leibold MA (1995) The niche concept revisited: mechanistic models and community context. Ecology 76: 1371–1382.

[4] Real LA, Levin SA (1991) Theoretical advances: the role of theory in the rise of modern ecology In: Real LA, Brown JH, editors. Foundations of ecology: classic papers with commentaries. Chicago, IL: University of Chicago Press pp. 177–191.

[5] Hubbell SP (2001) The unified neutral theory of biodiversity and biogeography (MPB-32) (Vol. 32). Princeton University Press. 448p.

[6] Newsome SD, Martínez del Río C, Bearhop S, Phillips DL (2007) A niche for isotopic ecology. Front Ecol Environ 5: 429–436.

[7] Holt RD (2009) Bringing the Hutchinsonian niche into the 21st century: Ecological and evolutionary perspectives. Proc Natl Acad Sci 106: 19659–19665. 10.1073/pnas.0905137106 19903876 PMC2780934

[8] Bearhop S, Thompson DR, Waldron S, Russell IC, Alexander G, Furness LW (1999) Stable isotopes indicate the extent of freshwater feeding by cormorants *Phalacrocorax carbo* shot at inland fisheries in England. J Appl Ecol 36: 75–84.

[9] Bocher P, Cherel Y, Hobson KA (2000) Complete trophic segregation between South Georgian and common diving petrels during breeding at Iles Kerguelen. Mar Ecol-Prog Ser 208: 249–264.

[10] Awkerman JA, Hobson KA, Anderson DJ (2007) Isotopic (δ^15^N and δ^13^C) evidence for intersexual foraging differences and temporal variation in habitat use in Waved Albatrosses. Can J Zool 85: 273–279.

[11] Bearhop S, Adams CE, Waldron S, Fuller RA, MacLeod H (2004) Determining trophic niche width: a novel approach using stable isotope analysis. J Anim Ecol 73: 1007–1012.

[12] Cherel Y, Hobson K A (2007) Geographical variation in carbon stable isotope signatures of marine predators: a tool to investigate their foraging areas in the Southern Ocean. Mar Ecol Prog Ser 329: 281–287.

[13] Matthews B, Mazumder A (2004) A critical evaluation of intrapopulation variation of delta C-13 and isotopic evidence of individual specialization. Oecologia 140: 361–371. 15118902 10.1007/s00442-004-1579-2

[14] Semmens BX, Ward EJ, Moore JW, Darimont CT (2009) Quantifying inter-and intrapopulation niche variability using hierarchical Bayesian stable isotope mixing models. PloS one 4: e6187 10.1371/journal.pone.0006187 19587790 PMC2704373

[15] Barrett K, Anderson WB, Wait DA, Grismer LL, Polis GA, Rose MD (2005) Marine subsidies alter the diet and abundance of insular and coastal lizard populations. Oikos 109: 145–153.

[16] Bolnick DI, Svanbäck R, Fordyce JA, Yang LH, Davis JM, Hulsey CD (2003) The ecology of individuals: incidence and implications of individual specialization. Am Nat 161: 1–28. 12650459 10.1086/343878

[17] DeNiro MJ, Epstein S (1981) Influence of diet on the distribution of nitrogen isotopes in animals. Geochim Cosmochim Acta 45: 341–351.

[18] Kelly JF (2000) Stable isotopes of carbon and nitrogen in the study of avian and mammalian ecology. Can J Zool 78: 1–27.

[19] Reich KJ, Bjorndal KA, del Rio CM (2008) Effects of growth and tissue type on the kinetics of 13C and 15N incorporation in a rapidly growing ectotherm. Oecologia 155: 651–663. 10.1007/s00442-007-0949-y 18188602

[20] Rosenblatt AE, Heithaus MR (2013) Slow isotope turnover rates and low discrimination values in the American alligator: implications for interpretation of ectotherm stable isotope data. Physiol Biochem Zool 86: 137–148. 10.1086/668295 23303328

[21] Vander Zanden HB, Bjorndal KA, Reich KJ, Bolten AB (2010) Individual specialists in a generalist population: results from a long-term stable isotope series. Biol Lett 6: 711–714. 10.1098/rsbl.2010.0124 20335202 PMC2936143

[22] Magnusson WE, Carmozina de Araújo M, Cintra R, Lima AP, Martinelli LA, Sanaiotti TM, et al (1999) Contributions of C3 and C4 plants to higher trophic levels in an Amazonian savanna. Oecologia 119: 91–96. 28308164 10.1007/PL00008821

[23] Magnusson WE, Lima AP, Faria AS, Victoria RL, Martinelli LA (2001) Size and carbon acquisition in lizards from Amazonian savannah: evidence from isotope analysis. Ecology 82: 1772–1780.

[24] Warne RW, Pershall AD, Wolf BO (2010a) Linking precipitation and C3-C4 plant production to resource-dynamics in higher-trophic-level consumers. Ecology 91: 1628–1638. 20583705 10.1890/08-1471.1

[25] Boecklen WJ, Yarnes CT, Cook BA, Avis CJ (2011) On the use of stable isotopes in trophic ecology. Annu Rev Ecol Evol Syst 42: 411–440.

[26] Bostic DL (1966) Food and feeding behavior of the teiid lizard, *Cnemidophorus hyperythrus beldingi* . Herpetologica 22: 23–31.

[27] Asplund KK (1967) Ecology of Lizards in the Relictual Cape Flora, Baja California. Am Midl Nat 77: 462–475.

[28] Karasov WH, Anderson RA (1984) Interhabitat differences in energy acquisition and expenditure in a Lizard. Ecology 65: 235–247.

[29] Galina P (1994) Estudio comparativo de tres especies de lacertilios en un matorral desértico de la Región del Cabo, B.C.S., México. Tesis de Maestría. CIBNOR, La Paz, BCS, Mexico.

[30] Grenot CJ, Galina-Tessaro P, Alvarez-Cárdenas S (1995) Field metabolism of lizards from lower altitude regions of Baja California Sur (Mexico). Amphib-Reptil 16: 11–23.

[31] Polis GA (1991) Complex Trophic Interactions in Deserts: An Empirical Critique of Food-Web Theory. Am Nat. 138:123–155.

[32] Bostic DL (1965) Home range of the teiid lizard *Cnemidophorus hyperythrus beldingi* . Southwest Nat 10: 278–281.

[33] Peinado M, Alcaraz FDJ, Aguado I (1994) Fitogeografía de la península de Baja California, México. An Jard Bot Madrid 51: 255–277.

[34] García E (1998) Climas (Clasificación Köppen, modificado por García). Escala 1:1000,000 Comisión Nacional para el Conocimiento y Uso de la Biodiversidad (CONABIO) México.

[35] Roberts NC (1989) Baja California plant field guide Jolla La, California:Natural History Publishing Co. 309 p.

[36] O'Leary MH (1988) Carbon isotopes in photosynthesis. Bioscience 38: 328–336.

[37] Castillo LP, Hatch KA (2007) Fasting increases δ15N‐values in the uric acid of *Anolis carolinensis* and *Uta stansburiana* as measured by nondestructive sampling. Rapid Commun Mass Sp 21: 4125–4128. 18023076 10.1002/rcm.3305

[38] Langkilde T, Shine R (2006) How much stress do researchers inflict on their study animals? A case study using a scincid lizard, *Eulamprus heatwolei* . J Exp Biol 209: 1035–1043. 16513929 10.1242/jeb.02112

[39] Lin Z-H, Ji X (2005) Partial tail loss has no severe effects on energy stores and locomotor performance in a lacertid lizard, *Takydromus septentrionalis* . J Comp Physiol B 175: 567–573. 16133493 10.1007/s00360-005-0017-z

[40] Logan JM, Jardine TD, Miller TJ, Bunn SE, Cunjak RA, Lutcavage ME (2008) Lipid corrections in carbon and nitrogen stable isotope analyses: comparison of chemical extraction and modelling methods. J Anim Ecol 77: 838–846. 10.1111/j.1365-2656.2008.01394.x 18489570

[41] Layman CA, Arrington DA, Montaña CG, Post DM (2007) Can stable isotope ratios provide for community-wide measures of trophic structure? Ecology 88: 42–48. 17489452 10.1890/0012-9658(2007)88[42:csirpf]2.0.co;2

[42] Syväranta J, Lensu A, Marjomäki TJ, Oksanen S, Jones RI (2013) An empirical evaluation of the utility of convex hull and standard ellipse areas for assessing population niche widths from stable isotope data. PloS One 8: e56094 10.1371/journal.pone.0056094 23405254 PMC3566058

[43] Jackson MC, Donohue I, Jackson AL, Britton JR, Harper DM, Grey J (2012) Population-level metrics of trophic structure based on stable isotopes and their application to invasion ecology. PloS One 7: e31757 10.1371/journal.pone.0031757 22363724 PMC3283663

[44] Jackson AL, Inger R, Parnell AC, Bearhop S (2011) Comparing isotopic niche widths among and within communities: SIBER–Stable Isotope Bayesian Ellipses in R. J Anim Ecol 80: 595–602. 10.1111/j.1365-2656.2011.01806.x 21401589

[45] Little RC, Milliken GA, Stroup WW, Wolfinger RD, Schabenberger O (2006) SAS for mixed models, Cary, NC SAS Institute Inc.

[46] Rousseeuw P J, Leroy DAM (1987) Robust regression and outlier detection New York: John Wiley & Sons, Inc. 352 p.

[47] Insightful Corporation (2001) S-Plus 6 for Windows guide to statistics Washington: Insightful Corporation, Seattle. 622 p.

[48] Wiegand T, Moloney KA (2014) Handbook of spatial point-pattern analysis in ecology Chapman & Hall / CRDC applied environmental statistics CRC Press / Taylor & Francis, Boca Raton, FL, 538 p.

[49] Delaney KS, Riley SP, Fisher RN (2010) A rapid, strong, and convergent genetic response to urban habitat fragmentation in four divergent and widespread vertebrates. PLoS One 5: e12767 10.1371/journal.pone.0012767 20862274 PMC2940822

[50] Márquez-Ferrando R, Santos X, Pleguezuelos JM, Ontiveros D (2009) Bioaccumulation of heavy metals in the lizard *Psammodromus algirus* after a tailing-dam collapse in Aznalcóllar (Southwest Spain). Arch Environ Contam Toxicol 6: 276–285. 10.1007/s00244-008-9189-3 18587604

[51] Gillespie JH (2013) Application of stable isotope analysis to study temporal changes in foraging ecology in a highly endangered amphibian. PloS One, 8: e53041 10.1371/journal.pone.0053041 23341920 PMC3546114

[52] Martínez del Río C, Wolf N, Carleton SA, Gannes LZ (2009) Isotopic ecology ten years after a call for more laboratory experiments. Biol Rev 84: 91–111. 10.1111/j.1469-185X.2008.00064.x 19046398

[53] Gannes LZ, Martínez del Rio C, Koch P (1998) Natural abundance variations in stable isotopes and their potential uses in animal physiological ecology. Comp. Biochem Physiol 119A: 725–737. 10.1016/s1095-6433(98)01016-2 9683412

[54] Layman CA, Araujo MS, Boucek R, Hammerschlag‐Peyer CM, Harrison E, Jud ZR et al (2012) Applying stable isotopes to examine food‐web structure: an overview of analytical tools. Biol Rev 87: 545–562. 10.1111/j.1469-185X.2011.00208.x 22051097

[55] Warne RW, Gilman CA, Wolf BO (2010b) Tissue‐Carbon Incorporation Rates in Lizards: Implications for Ecological Studies Using Stable Isotopes in Terrestrial Ectotherms. Physiol Biochem Zool 83: 608–617. 10.1086/651585 20441446

[56] Willson JD, Winne CT, Pilgrim MA, Romanek CS, Gibbons JW (2010) Seasonal variation in terrestrial resource subsidies influences trophic niche width and overlap in two aquatic snake species: a stable isotope approach. Oikos 119: 1161–1171.

[57] Comas M, Escoriza D, Moreno-Rueda G (2014) Stable isotope analysis reveals variation in trophic niche depending on altitude in an endemic alpine gecko. Basic Appl Ecol 15: 362–369.

[58] Tieszen LL, Ode DJ, Barnes PW, Bultsma PM (1980) Seasonal variation in C_3_ and C_4_ biomass at the Ordway Prairie and selectivity by bison and cattle. In Kucera CL, editor. Proceedings of the 7th North American Prairie Conference. Missouri: Springfield. pp. 165-I 74.

[59] Ambrose SH, DeNiro MJ (1986) The isotopic ecology of East African mammals. Oecologia 69: 395–406. 28311342 10.1007/BF00377062

[60] Tieszen LL (1991) Natural variations in the carbon isotope values of plants: Implications for archaeology, ecology and paleoecology. J Archaeol Sci 18: 227–248.

[61] Wolf BO, Martínez del Río C (2003) How important are columnar cacti as sources of water and nutrients for desert consumers? A review. Isotopes Environ Health Stud 39: 53–67. 12812255 10.1080/1025601031000102198

[62] Sims PL, Singh JS, Lauenroth WK (1978) The Structure and Function of Ten Western North American Grasslands: I. Abiotic and Vegetational Characteristics. J Ecol 66 1: 251–285.

[63] Fleming TH, Nuñez RA, Lobo-Sternberg LS (1993) Seasonal changes in the diets of migrant and non-migrant nectarivorous bats as revealed by carbon stable isotope analysis. Oecologia 94: 72–75. 28313861 10.1007/BF00317304

[64] Wolf BO, Martínez del Río C, Babson J (2002) Stable isotopes reveal that saguaro fruit provides different resources to two desert dove species. Ecology 83: 1286–1293.

[65] Shreve F (1942) The desert vegetation of North America. Bot Rev 8: 195–246.

[66] Mann J (1969) Cactus feeding insects and mites. Bull. US Natn. Mus. 256: 1–158.

[67] Vázquez-Reyes CJ (2006) Patrones de uso de microhábitat de *Aspidoscelis hyperythra* en Baja California Sur. Implicaciones para su distribución y conservación. Ms. Thesis. Centro de Investigaciones Biológicas de Noroeste. La Paz, Baja California Sur (México).

[68] Ambrose SH (1991) Effects of diet, climate and physiology on nitrogen isotope abundances in terrestrial food webs. J Archaeol Sci 18: 293–317.

[69] Gröcke DR, Bocherens H, Mariotti A (1997) Annual rainfall and nitrogen-isotope correlation in macropod collagen: application as a palaeoprecipitation indicator. Earth Planet Sci Lett 153: 279–285.

[70] Hobson KA, Alisauskas RT, Clark RG (1993) Stable-nitrogen isotope enrichment in avian tissues due to fasting and nutritional stress: implications for isotopic analysis of diet. Condor 95: 388–394.

[71] McCue MD, Pollock ED (2008) Stable isotopes may provide evidence of starvation in reptiles. Rapid Commun Mass Spectrom 22: 2307–2314. 10.1002/rcm.3615 18613003

[72] Heaton THE, Vogel JC, von la Chevallerie G, Collett G (1986) Climate influence on the isotopic composition of bone nitrogen. Nature (Lond.) 322: 822–823.

[73] Polis GA, Sanchez-Piñero F, Stapp PT, Anderson WB, Rose MD (2004) Trophic flows from water to land: marine input affects food webs of islands and coastal ecosystems worldwide In: Polis GA, Power ME, Huxel G, editors. Food Webs at the Landscape Level. Chicago: University of Chicago Press pp. 200–216.

[74] Sealy JC, van der Merwe NJ, Lee Thorp JA, Lanham JL (1987) Nitrogen isotopic ecology in southern Africa: implications for environmental and dietary tracing. Geochim Cosmochim Acta 51: 2707–2717.

[75] Thackeray JF, van der Merwe NJ, Lee Thorp JA, Sealy JC (1993) Relationships between stable carbon and nitrogen isotope ratios in bone collagen of African ungulates. South Afr J Zoology 89: 458–459.

[76] Araujo MS, Bolnick DI, Layman CA (2011). The ecological causes of individual specialisation. Ecol Lett 14: 948–958. 10.1111/j.1461-0248.2011.01662.x 21790933

[77] INEGI (Instituto Nacional de Estadística, Geografía e Informática) (2003) Carta de uso actual del suelo y vegetación. Serie III. Mexico DF, Mexico.

