# Supplementary figures and images for: Isotopic Niche Variation in a Higher Trophic Level Ectotherm: Highlighting the Role of Succulent Plants in Desert Food Webs
Source: PLoS One. 2015 May 14;10(5):e0126814. doi: 10.1371/journal.pone.0126814 (PMC4431868; doi:10.1371/journal.pone.0126814)

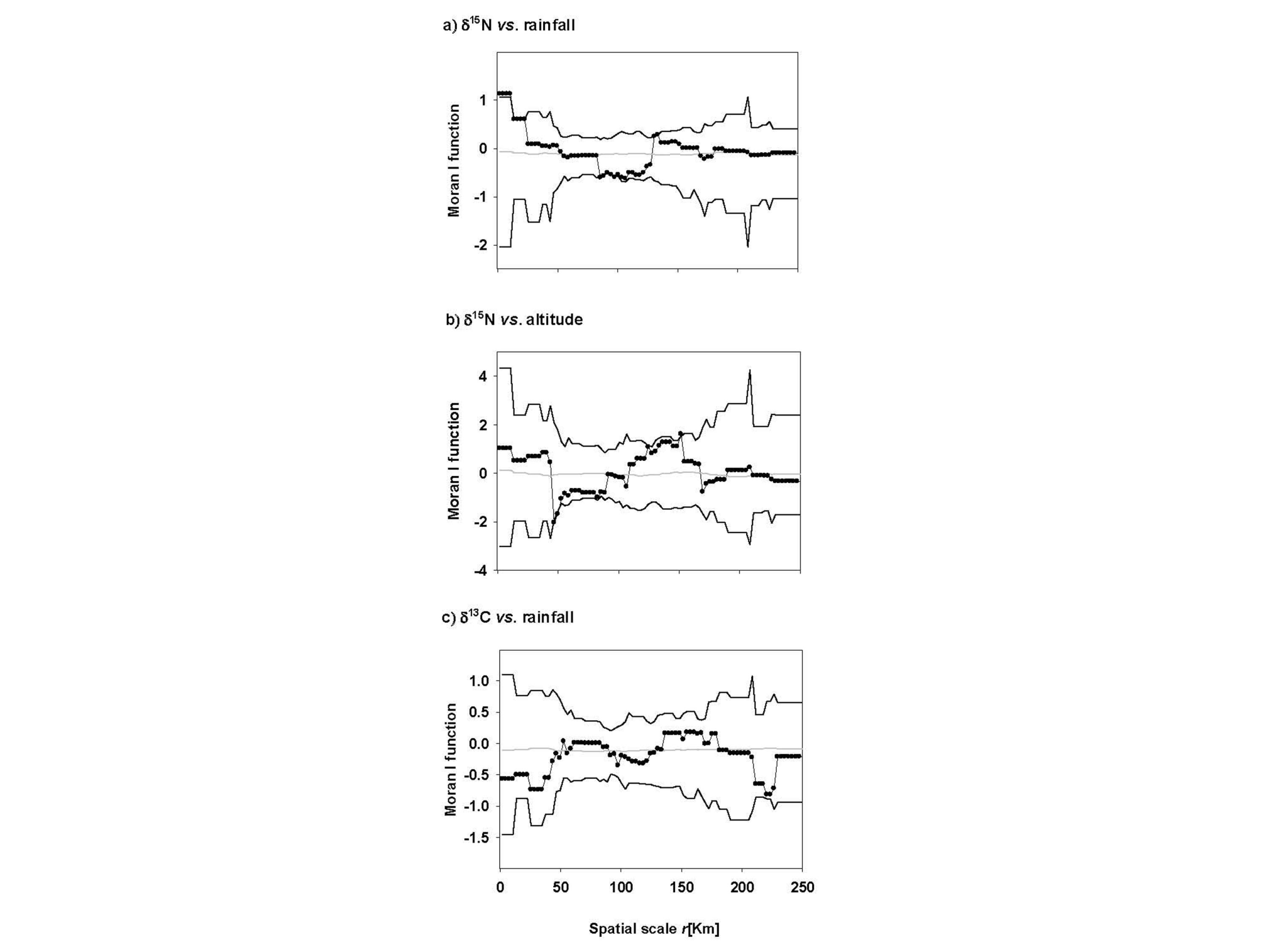

Supplement: S1 Fig — A), δ15N and rainfall; (B), δ15N and altitude; (C), δ13C and rainfall. (TIF) [file pone.0126814.s001.tif]

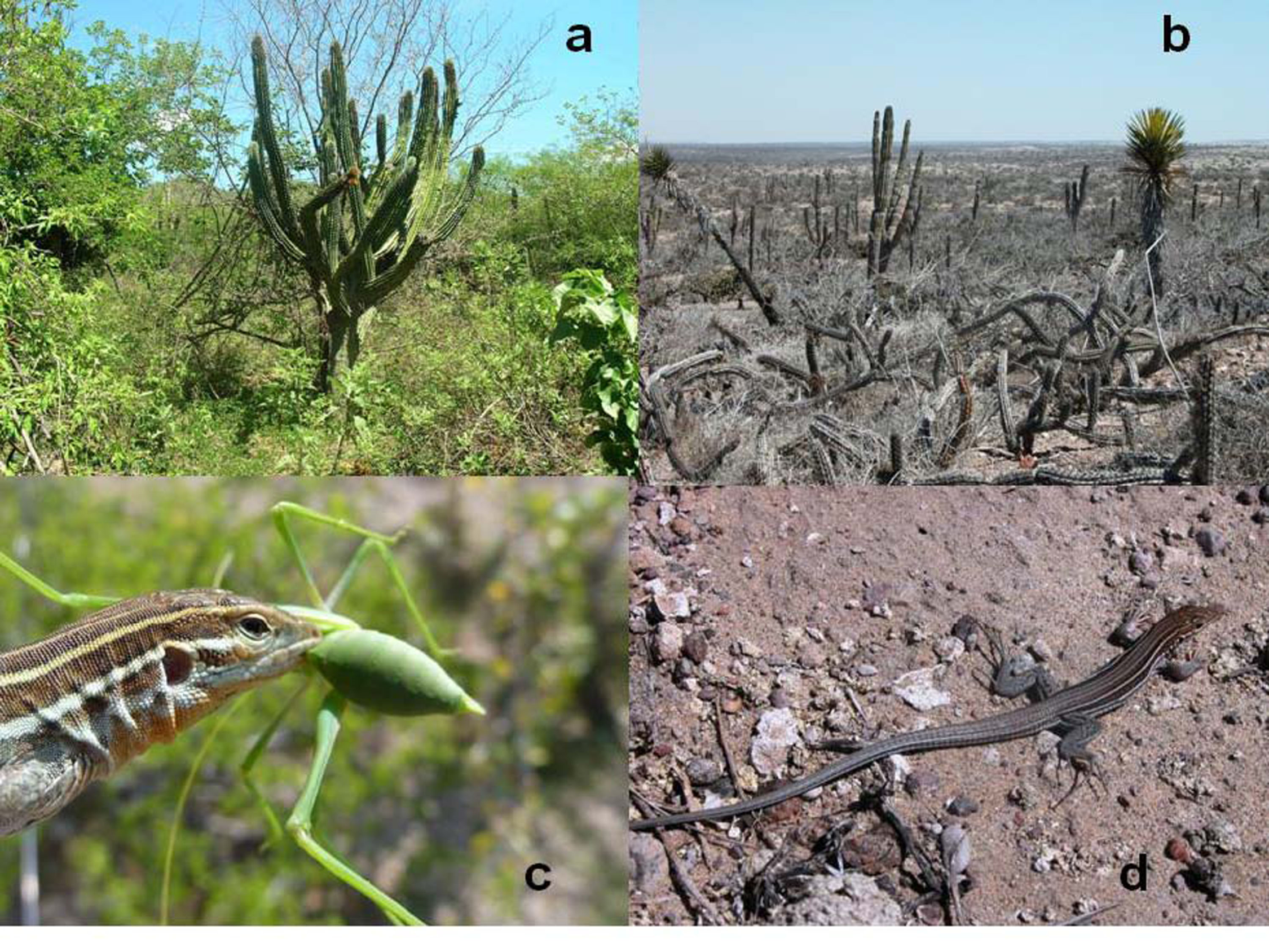

Supplement: S2 Fig — (A) Landscape at a subtropical shrubland in the Southern of the study area; (B) Landscape at the dry desert in the North of the study area, with abundance of succulents; (C) a Whiptail capturing an Orthoptera in the South; (D) a whiptail on the Northern desert. All the pictures by M. Delibes. (TIF) [file pone.0126814.s002.tif]
