# Supplementary material for: Isotopic Niche Variation in a Higher Trophic Level Ectotherm: Highlighting the Role of Succulent Plants in Desert Food Webs
Source: PLoS One. 2015 May 14;10(5):e0126814. doi: 10.1371/journal.pone.0126814 (PMC4431868; doi:10.1371/journal.pone.0126814)
Supplement: S1 Table — (DOC) [file pone.0126814.s003.doc]

**S1 Table .** Values of δ^15^N and δ^13^C in sampled C_3_, CAM and C_4_ plants collected at different localities of the study area in southern Baja California (Mexico).

|  | **Locality Coordinates** | **δ^15^N ‰ (Air-N_2_)** | **δ^13^C ‰ (V-PDB)** |
| --- | --- | --- | --- |
| **C3 plants** |  |  |  |
| *Adelia brandegeei* (Euphorbiaceae) | 23.2810N, 110.1358W | 6.74 | 24.00 |
| *Bursera hindsiana* (Burseraceae) | 25.8360N, 111.3337W | 13.09 | -27.82 |
| *Bursera hindsiana* (Burseraceae) | 23.3917N, 110.2383W | 13.24 | -24.52 |
| *Bursera microphylla* (Burseraceae) | 27.3650N, 112.2908W | 8.87 | -26.98 |
| *Bursera microphylla* (Burseraceae) | 25.8360N, 111.3337W | 6.71 | -29.89 |
| *Caesalpinia* sp. (Fabaceae) | 23.0350N, 109,7142W | 7.49 | -28.69 |
| *Parkinsonia florida* (Fabaceae) | 23.2810N, 110.1358W | 10.79 | -26.00 |
| *Cyrtocarpa edulis* (Anacardiaceae) | 24.2950N, 110.9683W | 1.83 | -28.20 |
| *Encelia* sp. (Asteraceae) | 27.3650N, 112.2908W | 13.29 | -28.13 |
| *Fouquieria diguetii* (Fouquieriaceae) | 25.8360N, 111.3337W | 7.40 | -26.93 |
| *Fouquieria diguettii* (Fouquieriaceae) | 27.3650N, 112.2908W | 8.61 | -23.67 |
| *Jatropha cinerea* (Euphorbiaceae) | 27.6800N, 113.4067W | 15.25 | -24.21 |
| *Jatropha cuneata* (Euphorbiaceae) | 23.2810N, 110.1358W | 7.28 | -25.42 |
| *Jatropha cuneata* (Euphorbiaceae) | 27.3650N, 112.2908W | 6.58 | -26.40 |
| *Larrea tridentata* (Zygophyllaceae) | 24.2950N, 110.9683W | 5.14 | -23.82 |
| *Lycium* sp. (Solanaceae) | 23.2810N, 110.1358W | 11.22 | -27.08 |
| *Maytenus phyllanthoides* (Celastraceae) | 23.2810N, 110.1358W | 5.78 | -26.95 |
| *Olneya tesota* (Fabaceae) | 27.3650N, 112.2908W | 6.64 | -26.24 |
| *Prosopis glandulosa* (Fabaceae) | 25.0160N, 111.6660W | 2.59 | -28.35 |
| *Solanum hindsianum* (Solanaceae) | 23.3917N, 110.2383W | 12.98 | -27.54 |
| *Tecoma stans* (Bignoniaceae) | 24.3717N, 111.0953W | 10.87 | -24.72 |
| Average |  | 8.68 | -26.43 |
| **CAM plants** |  |  |  |
| *Agave* sp. (Asparagaceae) | 23.9923N, 109.8242W | 14.80 | -14.76 |
| *Ferocactus diguetii* (Cactaceae) | 23.9923N, 109.8242W | 10.90 | -12.52 |
| *Ferocactus diguetii* (Cactaceae) | 25.0555N, 110.8838W | 15.78 | -13.41 |
| *Lophocereus schottii* (Cactaceae) | 23.9923N, 109.8242W | 17.78 | -14.94 |
| *Lophocereus schottii* (Cactaceae) | 23.9923N, 109.8242W | 9.82 | -15.64 |
| *Lophocereus schottii* (Cactaceae) | 25.0555N, 110.8838W | 15.75 | -14.55 |
| *Lophocereus schottii* (Cactaceae) | 25.3033N, 110.9504W | 15.52 | -15.77 |
| *Mammillaria* sp. (Cactaceae) | 23.9923N, 109.8242W | 10.44 | -14.98 |
| *Mammillaria* sp. (Cactaceae) | 25.3033N, 110.9504W | 9.10 | -14.73 |
| *Mammillaria* sp. (Cactaceae) | 25.3033N, 110.9504W | 8.12 | -14.20 |
| *Cylindropuntia cholla* (Cactaceae) | 23.9923N, 109.8242W | 12.54 | -14.98 |
| *Cylindropuntia cholla* (Cactaceae) | 25.0555N, 110.8838W | 14.70 | -14.27 |
| *Cylindropuntia cholla* (Cactaceae) | 25.3033N, 110.9504W | 8.63 | -14.76 |
| *Cylindropuntia molesta* (Cactaceae) | 25.0555N, 110.8838W | 11.77 | -12.06 |
| *Cylindropuntia molesta* (Cactaceae) | 25.3033N, 110.9504W | 6.36 | -11.72 |
| *Pachycereus pringlei* (Cactaceae) | 23.9923N, 109.8242W | 12.15 | -13.41 |
| *Pachycereus pringlei* (Cactaceae) | 25.0555N, 110.8838W | 10.60 | -12.03 |
| *Pachycereus pringlei* (Cactaceae) | 25.3033N, 110.9504W | 15.09 | -12.70 |
| *Euphorbia lomelii* (Euphorbiaceae) | 25.3033N, 110.9504W | 8.93 | -14.01 |
| *Stenocereus gummosus* (Cactaceae) | 23.9923N, 109.8242W | 16.68 | -14.24 |
| *Stenocereus gummosus* (Cactaceae) | 25.0555N, 110.8838W | 9.28 | -12.84 |
| *Stenocereus gummosus* (Cactaceae) | 25.3033N, 110.9504W | 10.23 | -15.72 |
| *Stenocereus thurberi* (Cactaceae) | 23.9923N, 109.8242W | 11.67 | -15.32 |
| *Stenocereus thurberi* (Cactaceae) | 25.0555N, 110.8838W | 14.35 | -13.26 |
| *Stenocereus thurberi* (Cactaceae) | 25.3033N, 110.9504W | 8.63 | -12.99 |
| Average |  | 11.86 | -13.91 |
| **C4 plants** |  |  |  |
| Gramineae | 24.4970N, 110.7250W | 7.32 | -14.86 |
